# Magnetic motor evoked potentials of cervical muscles in horses

**DOI:** 10.1186/s12917-018-1620-z

**Published:** 2018-09-24

**Authors:** Joke Rijckaert, Bart Pardon, Luc Van Ham, Philip Joosten, Gunther van Loon, Piet Deprez

**Affiliations:** 1 Department of Large Animal Internal Medicine, Faculty of Veterinary Medicine, Ghent University, Salisburylaan 133, 9820 Merelbeke, Belgium; 2 Department of Obstetrics, Reproduction and Herd Health, Faculty of Veterinary Medicine, Ghent University, Salisburylaan 133, 9820 Merelbeke, Belgium; 3 Small Animal Department, Faculty of Veterinary Medicine, Ghent University, Salisburylaan 133, 9820 Merelbeke, Belgium

**Keywords:** EMG, Neurologic test, Spinal ataxia, Surgery, Transcranial magnetic stimulation

## Abstract

**Background:**

When surgical treatment of cervical vertebral malformation is considered, precise localization of compression sites is essential, but remains challenging. Magnetic motor evoked potentials (mMEP) from paravertebral muscles are useful in localizing spinal cord lesions, but no information about cervical muscle mMEP in horses is available yet. Therefore, the aim of this study was to determine the possibility, normal values, inter- and intra-observer agreement and factors that have an effect on cervical mMEP in healthy horses.

**Methods:**

Transcranial magnetic stimulation was performed on 50 normal horses and 4 (2 left, 2 right) muscle responses were recorded at the middle of each cervical vertebra (C1-C7) and additionally just caudal to C7 to evaluate cervical nerves (Cn) Cn1 to Cn8. Latency time and amplitude of the recorded mMEP were defined by both an experienced and an unexperienced operator.

**Results:**

Latency increased gradually from 14.2 ± 1.38 ms for Cn3 to 17.7 ± 1.36 ms for Cn8, was significantly influenced by cervical nerve (*P* < 0.01), gender (*P* = 0.02) and height (*P* = 0.03) and had a good intra-observer agreement. The smallest mean amplitude (4.35 ± 2.37 mV) was found at Cn2, the largest (5.99 ± 2.53 mV) at Cn3. Amplitude was only significantly influenced by cervical nerve (*P* < 0.01) and had a low intra-observer agreement. No significant effect of observer on latency (*P* = 0.88) or amplitude (*P* = 0.99) measurements was found.

**Conclusion:**

mMEP of cervical muscles in normal horses are easy to collect and to evaluate with limited intra- and inter-observer variation concerning amplitude and should be investigated in future studies in ataxic horses to evaluate its clinical value.

## Background

In human patients with compressive myelopathy, spinal cord vascular disorders, myelitis or spinal cord injury, medical diagnostic imaging provides detailed information, but often shows compression in asymptomatic lesions [1–4]. Also in horses, many studies highlight the controversy, difficulties and limitations of cervical radiography, myeolography, computed tomography (CT), magnetic resonance imaging (MRI) and myeloscopy to diagnose spinal cord disease. Sensitivities (47–50%) and specificities (70–78%) of cervical radiographs and sagittal ratio calculations are too low for adequate diagnosis of spinal cord compression [5, 6] and variation between observers is high [7]. Myelography also has a low sensitivity (43–85%) and additionally requires general anesthesia and intrathecal contrast injection [6, 8]. Most CT and MRI scanners can only image the cranial cervical spinal cord because of the limited diameter of the CT and MRI gantry. This is an important limitation since 37–54% of CVM lesions occur in the caudal (C5-C7) cervical vertebral column [6, 8]. Furthermore, no flexion or extension of the neck is possible in CT or MRI scanning [9–11]. Cervical vertebral canal endoscopy is not routinely performed as there is a high risk of complications associated with entering the spinal canal or due to neck movement during the procedure. In addition, the visual assessment of subarachnoid space narrowing may not be reliable in cases with mild to moderate stenosis [12].

On the other hand, non-infectious spinal ataxia and paresis is an important issue in horses. Major causes include trauma, neoplasia or equine degenerative myeloencephalopathy, but in European horses, spinal ataxia is most commonly caused by cervical vertebral malformation (CVM). CVM is a stenosis of the cervical vertebral canal with static or dynamic compression of the spinal cord [13] commonly seen in young, rapidly growing thoroughbreds and in warmblood horses [5, 6, 14]. Thoroughbreds (6 months–2 years) are prone to a dynamic compressive form (type 1), while the static form (type 2) typically affects older warmblood horses in the caudal cervical region and is caused by osteoarthritic enlargement of the cervical articular processes [5, 15].

Hoffman and Clark [16] described that some thoroughbreds (about 30%) were able to race at least once after CVM diagnosis and non-surgical management, but if bony malformations or soft tissue proliferations exist, neurological deficits remain present [13] and many horses are euthanized [6, 16]. Surgical treatment can improve prognosis: Moore, Reed [17] and Walmsley [18] described that 45–60% of the patients who underwent vertebral body fusion returned to use, but the clinical response to surgery depended strongly on the ability to identify all compressed sites [17, 18]. Although this precise localization is essential, it still remains challenging because of the limitations of medical imaging. Therefore, it is essential to correlate results of medical imaging with neurophysiological examinations [2, 19].

A highly accurate diagnostic test with a very high sensitivity in human patients is transcranial magnetic stimulation which evokes synchronized descending volleys in corticospinal pathways: magnetic motor evoked potentials (mMEP) [20]. MMEP with prolonged latencies indicate pathological slowing of the conduction through the corticospinal tract in a non-invasive way [21]. The test might be contra-indicated in epileptic patients and in patients with pacemakers or implanted metal structures in the brain but the incidence of side effects is low. By using specific muscles to record mMEP, such as paravertebral muscles, and by determination of central and peripheral motor conduction time, exact localization of lesions and distinction between central and peripheral neural pathology is possible in human patients [2, 22]. In ataxic or paralytic horses, lesions have already grossly been localized using mMEP conductions into thoracic and pelvic limbs [23–25]. Best results are obtained after sedation with detomidine (1 mg/100 kg) and buprenorphine (0.24 mg/100 kg) and stimulation with a round 70 mm diameter coil placed high on the frontal region of the horse, with a maximal output of the stimulator (maximal magnetic field of 4 Tesla) [26–28]. Coil current had no significant effect on latency values and no adverse effects have been reported [26]. Abnormal thoracic and pelvic limb muscle mMEP latency and amplitude values suggest cervical spinal cord disease [23] while pelvic muscle abnormalities alone, occur with thoracic or thoracolumbar pathology. However, no differentiation between etiology or central or peripheral lesions can be made [25]. Of course, it would be interesting to localize the lesion more precisely in horses, just like in human medicine, using cervical paravertebral muscles mMEP. These muscles are situated close to the vertebral column making the peripheral component of the nervous system small. This means that, if spinal root compression is absent, these cervical muscle mMEP will approximate the central motor conduction time, making distinction between central and peripheral pathology possible. However, this paravertebral muscles examination is currently unexplored in horses. Therefore, the aim of this study was to investigate whether paravertebral muscle mMEP recording is possible in horses and, if so, to determine normal values, inter- and intra-observer agreement and factors that might have an influence on mMEP recordings of paravertebral cervical muscles in healthy horses.

## Methods

### Animals

To determine reference values for mMEP in cervical muscles of horses, sample size was set at 50 as recommended [29]. Healthy horses (36 mares, 12 geldings and 2 stallions; 35 warmbloods, 11 trotters, 1 pony, 1 Friesian, 1 Arabian and 1 Andalusian) were conveniently selected. Thirty horses were owned by the faculty of veterinary medicine of Ghent University as laboratory animals, the other 20 horses were recipient mares for embryo transfers loaned by Keros plc. All horses returned to their owners 1 day after the test. The median age of the horses was 11.5 (range 3 to 22, 15 horses between 3 and 7, 14 between 8 and 12, 17 between 13 and 17 and 4 between 18 and 22) years, the median height160 (range 142 to 175) cm and median weight 553 (range 388 to 705) kg. All horses had a body condition score between 3/9 and 6/9. There were no significant age or height differences between males and females. The mean weight of male horses was significantly (*P* < 0.01) higher than the mean weight of the female horses (557 ± 79 versus 546 ± 76 kg). Rectal temperature was normal ranging from 37.1 to 37.9 °C. All horses were examined clinically and neurologically by a veterinarian with 3 years of experience in neurological examinations. The neurological evaluation form of Mayhew [30] was used as guideline. During the examination, special attention was paid to a normal mobility and the absence of any swelling of the neck. Only clinically and neurologically normal horses were included in this study.

### Magnetic stimulation and mMEP recording

Each horse was sedated intravenously with a combination of detomidine (Domidine, Eurovet Animal Health, 12 μg/kg bwt) and butorphanol (Dolorex, MSD Animal Health, 12 μg/kg bwt). After 5 min, the level of sedation of the horse was subjectively evaluated. Horses that were still alert and reactive to a hand clap, received an additional 6 μg/kg detomidine and 6 μg/kg butorphanol. For the magnetic stimulation and mMEP recording, the horses were all placed in the same examination room with an environmental temperature ranging from 18 to 24 °C. A magnetic stimulator (Magstim 200, The Magstim Company) and a round 70 mm coil were used to generate a maximal magnetic field of 4 Tesla at the coil surface. The coil was centered over the dorsal part of the frontal bone as described by Nollet, Van Ham [26] and maximal stimulus intensity (100%) was applied. The muscle responses were recorded by a standard electromyograph (EMG; Medelec Sapphire, Medelec) through needle electrodes at the level of the middle of each vertebra (C1-C7) and additionally just caudal to the 7th vertebra (C7) to evaluate cervical nerves (Cn) Cn1 to Cn8. The active needle electrode (disposable monopolar needle electrode, 37 mm, 26G, TECA Corporation) was placed as deep as possible (full length or until contact with the vertebral bone, when it was pulled back 2 mm) to reach the paravertebral *intertransversarii cervicii* muscles of C3-C7. The accessibility of these paravertebral muscles was confirmed with ultrasound based on the study of Berg, Nielsen [31]. At the level of the atlas and axis, the needle was placed as deep as possible in the *obliquus capitis cranialis* and *caudalis* muscle, as there are no paravertebral *intertransversarii* muscles at this level. The reference electrode (disposable monopolar needle electrode, 25 mm, 26G, TECA Corporation) was placed subcutaneously at the level of the active electrode. The ground electrode was always attached at the level of the tuber olecranon. Localization of the measurement points was done by palpating the transversal processes of the cervical vertebra and visually determining the middle between 2 sequential processes. At each vertebra, two responses were recorded at the left side and two at the right side of the horse.

Latency and amplitude were defined for each response. Latency (ms) was defined as the time between the stimulation and the onset of contraction, indicated by the first deflection from the baseline. Amplitude (mV) was defined as the difference between the two largest peaks of opposite polarity. All stimulations were carried out by observer 1. On all recorded mMEP, marker positioning for latency and amplitude were done independently by the same two operators. Observer 1 had 3 years of experience in neurological examinations and recording of mMEP, observer 2 had no experience.

### Statistics

Data were entered on a spreadsheet (Excel, Microsoft Corporation) and transferred to SPSS 2.4 (IBM SPSS Statistics for Windows) for descriptive and statistical analysis. The continuous outcome variables latency and amplitude were checked for a normal distribution (Kolmogorov Smirnov test and inspection of Q-Q plots and histograms). To determine the 90% reference intervals (upper and lower bound) for latency and amplitude, an Excel add-in was used (Reference Value Advisor; Geffré et al., 2011) and the shortest latency time and the highest amplitude of the four stimulations per location per horse were used. The programme calculates untransformed, box transformed and non-parametric reference intervals and indicates which intervals comply best with the data set.

To determine factors which are significantly associated with latency and amplitude, linear mixed models were used with horse and cervical nerve as random effect to account for clustering of measurements within a horse. Predictors added to the model were cervical nerve [1–8], side (left or right), sedation dose, breed and the physiological parameters gender, age (years), body weight (kg) and withers height (cm). First, all parameters were tested univariably. Then, all parameters with *P* < 0.20 were retained for the final multivariable model, which was built stepwise backwards gradually, excluding non-significant factors. When predictor variables were highly correlated (Pearson correlation > 0.60), only the most significant variable was added to the model. Biologically relevant interactions of significant main effects were tested. For all significant categorical variables, pairwise comparisons were made using post-hoc tests with a Bonferroni correction to adjust for multiple comparisons.

Inter- and intra-observer agreements were determined for each location (four measurements per vertebra), using linear mixed model procedures and coefficients of variation (CV (%) = (SD/mean) × 100), respectively. CVs were calculated on horse level by taking the mean value of the CV per horse and on study population level by calculating the CV of the minimal latency and maximal amplitude values of 4 measurements per horse.

## Results

**Fig. 1:**
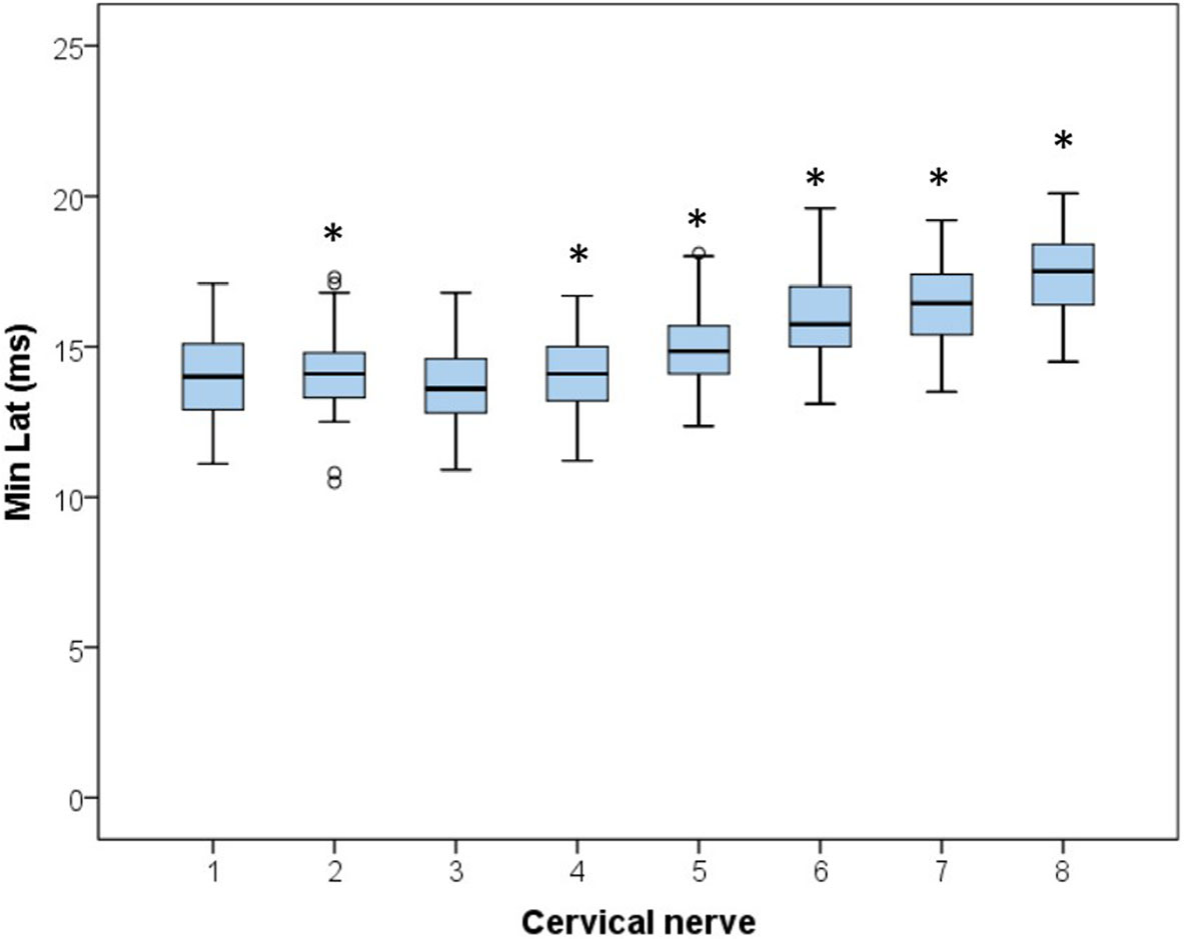
Boxplots for all latency values per cervical nerve (8 times *n* = 200). Significant differences (*P* < 0.01) from group 3, the group with the lowest latency values, are indicated by *, outliers are indicated by a circle

**Fig. 2:**
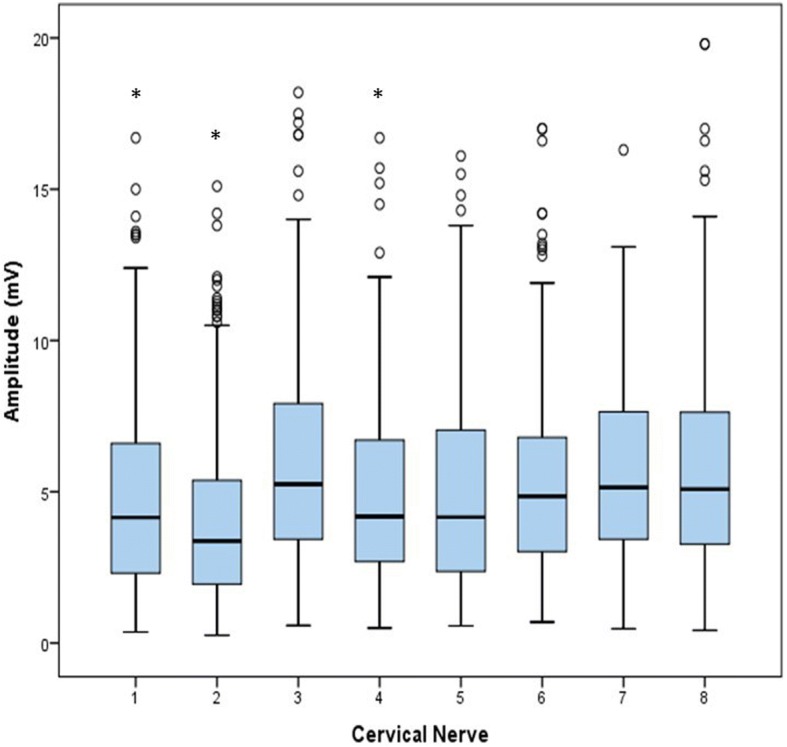
Boxplots for all amplitude values per cervical nerve (8 times *n* = 200). Analogous to latency, significant differences (*P* < 0.01) from group 3 are indicated by *, outliers are indicated by a circle

**Fig. 3:**
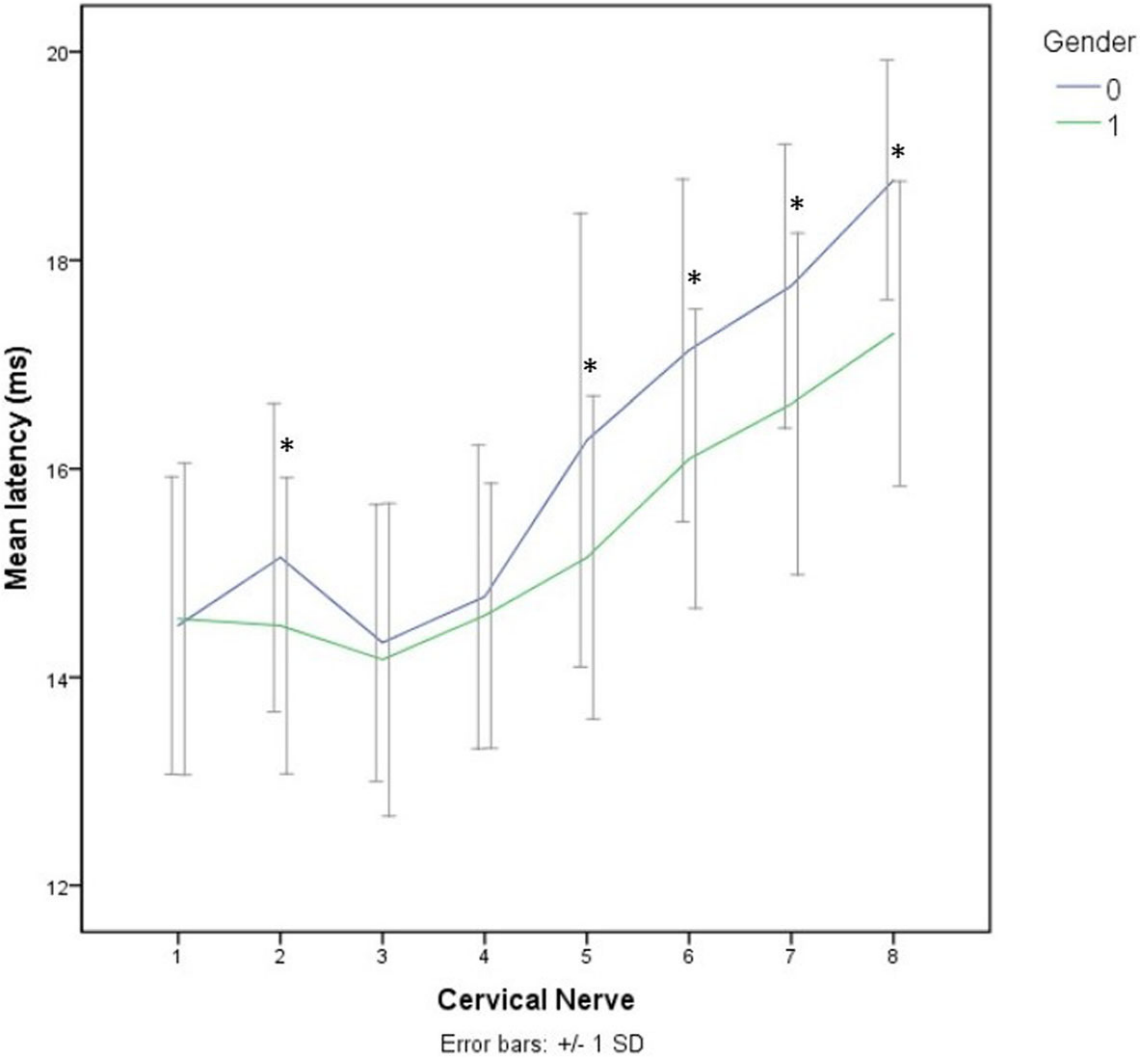
Interaction between gender (0 = male and 1 = female) and cervical nerve on latency. Significant differences (*P* < 0.05) are demonstrated by *

A total of 1600 stimulations were performed and all delivered measurable mMEP. Latency and amplitude recordings for each cervical nerve are displayed in Figs. 1 and 2 and descriptive statistics and calculated 90% reference intervals are presented in Table 1. Factors univariably associated with latency were cervical nerve (*P* < 0.001), gender (*P* = 0.07), age (*P* = 0.05), height (*P* < 0.001), weight (*P* < 0.001) and sedation dose (*P* < 0.001). In the final multivariable model for latency, only the factors cervical nerve (*P* < 0.01), gender (*P* = 0.02) and height (*P* = 0.03) remained significantly associated. Interactions between the different significant main effects were tested, but only the interaction between cervical nerve and gender was significant (*P <* 0.01) (Fig. 3). This interaction signifies a different effect of the cervical nerve location on latency time for males and females. In male horses Cn2, Cn5, Cn6, Cn7 and Cn8 had significantly (*P <* 0.01) longer latency times with mean differences of respectively 0.7, 1.1, 1.0, 1.1 and 1.5 ms compared to females. Irrespective of gender, latency increases gradually from 14.2 ms in Cn3 to 17.7 ms in Cn8 with significant differences between the different locations. Cn1 and Cn2 were not significantly different, but Cn2 had significantly longer latency values than Cn3 (Fig. 1).

**Table 1:** Mean observed values, standard deviations (SD), minimum (Min) and maximum (Max) values, and calculated reference intervals (RI) for latency (shortest latency of 4 observations per horse) and amplitude (maximum amplitude of 4 observations per horse) values for each cervical nerve in 50 healthy horses

	Cervical nerve	*n*	Mean	SD	Min	Max	90% RI
Latency (ms)	1	50	14.0	1.4	11.1	17.1	11.2–16.8
	2	50	14.1	1.4	10.5	17.3	10.6–17.2
	3	50	13.7	1.4	10.9	16.8	11.0–16.6
	4	50	14.1	1.2	11.2	16.7	11.4–16.6
	5	50	15.0	1.3	12.4	18.1	12.6–18.1
	6	50	15.9	1.5	13.1	19.6	13.2–19.4
	7	50	16.5	1.4	13.5	19.2	13.6–19.2
	8	50	17.4	1.3	14.5	20.1	14.6–20.1
Amplitude (mV)	1	50	8.2	3.7	0.9	16.7	1.2–16.2
	2	50	7.1	4.3	1.1	18.8	1.1–18.3
	3	50	9.3	4.0	2.0	18.2	2.2–18.0
	4	50	7.8	3.4	1.9	16.7	2.0–16.4
	5	50	8.4	3.9	1.5	16.1	1.6–15.9
	6	50	8.0	3.9	1.9	18.7	2.1–18.2
	7	50	8.3	2.9	1.8	16.3	2.1–15.4
	8	50	8.8	4.1	2.6	19.8	2.7–19.8

*n*, number of magnetic motor evoked potentials recorded

Data for amplitude needed to be log transformed to obtain a normal distribution. Factors univariably associated with amplitude were cervical nerve (*P* < 0.01) and sedation dose (*P* = 0.15). In the final multivariable model only cervical nerve was significant (*P* < 0.01). Amplitude was smallest (4.35 mV) in Cn2 and largest (5.99 mV) in Cn3. Cn2 had significantly different amplitude values compared to Cn3, Cn6, Cn7 and Cn8. Mean maximal amplitude for Cn3 was also significantly different from Cn1 and Cn4, and for Cn1 it was significantly different from Cn8.

Mixed models showed no significant effect of observer on latency (*P* = 0.88) or amplitude (*P* = 0.99). CV, on horse and on study population level, of latency and amplitude measurements for both observers are represented in Table 2.

**Table 2:** Coefficients of variation (CV (%) = SD/mean*100) per cervical nerve (Cn) of latency and amplitude on horse (mean of CVs calculated per horse) and study population (CV of minimal latency and maximum amplitude values per horse) level for observer 1 and 2

	Level	Observer	Cn1	Cn2	Cn3	Cn4	Cn5	Cn6	Cn7	Cn8	Mean
	Horse	1	3.10	3.00	2.70	3.19	3.76	2.48	3.16	2.24	2.79
		2	4.53	4.10	4.05	4.60	4.09	3.00	3.15	2.16	3.71
	Study population	1	9.71	9.87	10.14	8.81	8.76	9.31	8.36	7.67	9.08
		2	14.50	13.77	14.50	12.99	15.20	10.89	9.75	8.41	12.50
Amplitude	Horse	1	48.54	43.61	39.02	40.51	44.01	33.97	38.95	38.44	36.72
		2	49.61	44.57	39.95	40.39	43.18	36.96	39.31	38.44	41.55
	Study population	1	44.42	59.76	43.13	43.00	46.13	48.52	35.04	46.87	45.86
		2	57.26	60.32	43.23	44.22	46.83	46.02	36.78	61.21	49.48

## Discussion

Transcranial magnetic stimulation with registration of mMEP in cervical muscles is possible and easy to perform in horses, as all stimulations resulted in muscle responses from which latency and amplitude could be measured. All measurements of latency and amplitude were carried out by an experienced and an unexperienced observer. For latency, the variation per cervical nerve within a horse was very small for each observer, indicating good intra-observer repeatability. General CVs in this population of horses were larger but still small enough to state that latency was sufficiently repeatable [32, 33]. These findings correspond to the small variations in repeated recordings found in previous equine mMEP studies [23, 28, 34, 35]. Also inter-observer reproducibility was very good as mixed models did not show a significant observer effect. So, in this study, no significant observer effects could be demonstrated suggesting that latency is a reproducible parameter for cervical mMEP, even for clinicians without experience.

Latency values were influenced by height, cervical nerve and gender with a significant interaction between cervical nerve and gender. The significant effect of height was already found in horses [28] and humans [36]. The increasing neural length along the spinal cord, results in a progressive increase in latency [37]. This was confirmed in our study although no measurements of exact neural or neck length were performed. In general, the increase in latency was relatively small (3.3 ms) and started only from Cn3. Mean values for Cn1 and Cn2 were significantly larger than for Cn3. Obviously the anatomy of the atlas and axis region is totally different compared to the more caudal parts of the neck. So, at the level of C1 and C2, a different muscle, which might also be different in composition and innervation, was tested than more caudally along the neck. The influence of gender was not found previously in horses [28] nor in calves [38], but has been described in human subjects [39, 40], though most studies showed no gender effect once corrected for height [41]. In the present study, no differences in height were found between males and females, but a significant weight difference was present. The most likely hypothesis is that male horses have a higher level of muscularity (hence the higher body weight) which might result in a longer distance from stimulation to recording site and hence to longer latencies. The longer latency times in male horses became even clearer in the caudal, stronger muscled, parts of the neck supporting this hypothesis.

The reference intervals for latency were calculated and can be used in clinical situations in future. The 5–6 ms variation in latency values in normal horses is relatively large and probably caused by the large range in horse height in this study population and a sample size at the lower half of the recommended number for reference interval determinations [29]. In addition, subjective localization of the measuring points, based on palpation of the transverse processes and subjectively determining the middle of each vertebra, differences in local temperature or subclinical lesions may have contributed to some variation. However, this variation is probably of limited clinically importance in horses with severe cervical spinal cord disease as in such horses latency times commonly double [when recorded from caudal to the lesion] compared to normal values [23]. Only in subtle or subclinical cases, the variation might result in false negative results. This needs to be determined by validation studies in future, by including acute and chronic and mild and severe clinical case material.

With regard to amplitude, intra-observer repeatability was poor for both horse and general level. The large CVs for amplitude are a logical consequence of the large standard deviations compared to the mean values. Large CVs for amplitude were also found in normal human patients [34, 36], dogs [38, 42, 43] and previous mMEP studies in large animals [23, 26–28, 35, 44]. This large variation is possibly due to physical changes in stimulation (inter-trial variations: output of the stimulator, position of the coil, position of the needle electrodes) or neurophysiological differences in the patient (inter- and intra-individual variations: level of relaxation or voluntary contraction, excitability variations) [28, 29, 36]. However, these factors are difficult or impossible to control in a clinical setting. As a consequence, the calculated reference intervals are wide. In contrast, inter-observer reproducibility of amplitude measurements was good as mixed models showed no significant influence of observer. Thus, results for amplitude measurements are similar for experienced and non-experienced clinicians, but the clinical value of this parameter remains limited because of its high variation.

## Conclusion

Registration and measurement of latency and amplitude values from mMEP of cervical muscles in normal horses is easy, with limited intra- and inter-observer variation. The available reference values for healthy horses can be compared with values in horses with suspected cervical spinal cord disease to evaluate whether precise localization of lesions is possible.
